# Optimal Evaluation and Treatment of Cardiac Rehabilitation for Patients Following TAVR: A Personalized Program Based on The Five Prescriptions

**DOI:** 10.14336/AD.2025.0716

**Published:** 2026-10-04

**Authors:** Xue-Song Han, Gang Wang, Yong Miao, Zhi-Yuan Tian, Yong-Chao Zhang, Zong-Yan Li, Xiao-Jia Sun, Qiang Hu

**Affiliations:** ^1^No. 93279 Unit of the Chinese People’s Liberation Army, Dandong, China.; ^2^Department of Cardiology, Air Force Hospital of Western Theater Command, Chengdu, China.; ^3^The Outpatient Department of Sixth Garrison, General Hospital of Central Theater Command, Wuhan, China.; ^4^No. 95948 Unit of the Chinese People’s Liberation Army, Jiuquan, China.

**Keywords:** transcatheter aortic valve replacement, cardiac rehabilitation, evaluation, treatment, personal program

## Abstract

Transcatheter aortic valve replacement (TAVR) is widely recommended and applied among patients with severe aortic stenosis. However, considering the complicated situation of patients after TAVR, Cardiac rehabilitation (CR), as a well-established program to improve the outcome and quality of life, has not been underexploited among them yet. The purpose of this review is to discuss the current challenges of carrying out the post-TAVR CR program, compare different evaluation programs, and develop a relatively reasonable and standardised post-TAVR CR program based on “The Five Prescriptions” of *Guidelines for cardiovascular rehabilitation and secondary prevention in China*. Constructed from physical intervention, psychological intervention, nutritional intervention, and life management, the post-TAVR CR program targets a combination of universal and tailored approaches for patients.

## 1. Introduction

With remarkable medical evolution, transcatheter aortic valve replacement (TAVR) has been widely used in patients with severe aortic stenosis. The European Society of Cardiology (ESC) and the European Association for Cardio-Thoracic Surgery (EACTS) have given TAVR a Class Ⅰ recommendation in older patients (≥75 years) or in those who are at high risk or unsuitable for surgery [1]. Cardiac rehabilitation (CR) is a comprehensive, multidisciplinary program for secondary prevention of cardiovascular disease and is highly recommended for patients with cardiovascular disease. However, CR for patients with TAVR has not been systematically elaborated and has not even been recommended in the past guidelines [2]. Over time, more and more hospitals have tried to provide CR to improve patient outcomes and quality of life following TAVR. A recent meta-analysis showed that patients following TAVR improved their exercise capacity and functional independence by participating in exercise-based CR [3]. *Guidelines for cardiovascular rehabilitation and secondary prevention in China* presented a concept named “The five prescriptions” for CR, covering limiting-alcohol-and-quitting-smoking prescription, exercise prescription, drug prescription, psychological prescription, and nutritional prescription [4]. Based on “The Five Prescriptions”, this review aims to summarise the current state of cardiac rehabilitation among patients following TAVR and develop a new standardised program on CR for those patients.

## 2. Challenge of CR among patients undergoing TAVR

The efficacy of CR among patients undergoing TAVR may be easily influenced by the physiological, psychological, and social aspects. Moreover, CR is prone to be neglected by physicians and patients due to the unknown side effects of CR for these patients. As a result, CR adoption and application rates among patients undergoing TAVR remain low, although CR is increasingly encouraged by the current guidelines.

**Table 1:** Comorbidities and Postoperative Complications of Patients undergoing TAVR

Comorbidities	Prevalence
**Sarcopenia**	21.0%-70.2% ^[5]^
**Frailty**	50%-70% ^[6]^
**Malnutrition (Based on GNRI)**	43.9%^[7]^
**COPD**	28%-43% ^[8]^
**HF (LVEF<50%)**	23% ^[9]^
**Dementia**	5.0% ^[10]^

Postoperative Complications	Prevalence

**Permanent pacemaker implantation**	10.4% ^[11]^
**New-Onset Atrial Fibrillation (NOAF)**	9.9% ^[12]^
**Stroke**	1.4%-4.3% ^[11]^
**Infective endocarditis**	2.07% ^[13]^
**Aortic aneurysm**	0.849% ^[11]^
**Acute renal failure (Stage Ⅱ Ⅲ)**	0.72% ^[14]^
**Aortic dissection**	0.174% ^[11]^

TAVR: transcatheter aortic valve replacement; GNRI: geriatric nutritional risk index; COPD: chronic obstructive pulmonary disease; HF: heart failure; LVEF: left ventricular ejection fraction; NOAF: new-onset atrial fibrillation.

### 2.1 High rates of comorbidities and postoperative complications

Because of advancing age, patients undergoing TAVR have various kinds of comorbidities and postoperative complications (Table 1) [15, 16], which result in dire conditions for patients, such as disability and frailty. Comorbidities and postoperative complications affect physical and psychological function to different degrees. Exercise intolerance is widespread in patients with heart failure (HF), and it is more severe if they have atrial fibrillation (AF) [17, 18]. Patients with AF or HF also face a higher possibility of cognitive impairment than those without. Even HF and cognitive impairment aggravate each other [19]. Cardiopulmonary function is also confounded by diseases such as cognitive impairment, sarcopenia, and musculoskeletal weakness, which prevent CR participation in exercise training and health education [17, 20]. Moreover, the recurrence of diseases also influences the completion of CR in a way. In such circumstances, CR management of post-TAVR patients becomes quite complex.

### 2.2 Low levels of physical fitness and exercise capacity

Post-TAVR patients exhibit low levels of physical fitness and exercise capacity both before and during CR. Firstly, exercise tolerance is limited due to the valve stenosis itself. Although TAVR improves physical fitness to some extent, it does not entirely reverse decades-old deficits in exercise capacity and function [15]. The physical fitness and exercise capacity of post-TAVR patients are at a low level before and during CR. 6-minute walking test (6MWT) before CR was only 235.88 ± 69.36 m, and each exercise time is only 15-20 min [3, 21]. As for patients with frailty, one of the most common comorbidities among post-TAVR patients, Ushijima et al. [22] showed that usual walking speed, maximal grip strength, lower extremity strength, and peak work rate (WR) were significantly lower in comparison with non-frailty patients (1.06±0.20 vs. 1.24±0.19m/sec, *P*<0.001, 21.7±5.5 vs. 28.7±7.0kg, *P*<0.001, 0.37±0.09 vs. 0.50±0.18kgf/kg, *P*<0.001, 66±21 vs. 80±24watt, *P*<0.05). It is hard for patients undergoing TAVR, especially those with comorbidities, to undertake high-intensity exercise, even moderate-intensity exercise, for extended periods due to limited exercise capacity.

### 2.3 Low levels of cardiopulmonary function

VO_2_ peak is the “gold standard” for demonstrating the effect of the exercise component of CR, while heart rate variability (HRV) reflects the level of cardiovascular system adaptation [20, 23]. Generally, patients undergoing TAVR do not qualify for surgery or are at high risk just because of their limited cardiopulmonary function. In the research of Marcin et al. [24], each cumulative comorbidity or cardiovascular risk factor reduced VO_2_ peak by 1.7 ml/kg/min and 1.1 ml/kg/min, respectively. Among patients with stroke, one of the common postoperative complications of TAVR, HRV decreases and does not return to baseline within 30 minutes during exercise [20]. The problems above mean that a large group of patients undergoing TAVR have low adaptation in CR, and they may need a longer period of rehabilitation program than other types of patients.

### 2.4 Low levels of psychological function

Psychological factors affect the implementation of CR for patients with TAVR. Many elderly TAVR candidates suffer from mood disturbances due to their chronic disease or complications [25]. Patients with moderate depression (24% vs. 13%, *P*<0.001) or anxiety (32% vs. 23%, *P*<0.001) were more likely to face the CR dropout compared with patients with only normal to mild symptoms [26]. More direct evidence showed that depression was associated with adverse rehabilitation outcomes. As in the research of Bermudez et al. [27], the level of depression at the beginning of rehabilitation was negatively and significantly associated with exercise capacity as well as the emotional, physical, and social quality of life. However, the outcome of psychological function is able to be improved by timely psychological screening and reasonable psychological support [28].

### 2.5 Social-related risk factors

Due to their impaired physical function and exercise capacity, patients undergoing TAVR who were recommended for CR fear sustaining injury and are ashamed of their physical deficits [15]. In addition, they and their families fear that exercise or much more exercise would cause further heart damage [29], resulting in reduced CR participation or exercise training intensity regardless of whether post-TAVR patients are willing or forced to participate in the CR. Many patients will reduce CR participation more quickly because of the inconvenience of going to the rehabilitation center.

### 2.6 Lower adherence rate of CR

Post-TAVR patients, typically with various comorbidities, tend to have lower adherence to the CR. The participation rate was reported at <5% among adults aged 85 or older [20]. Patients with a higher prevalence of diabetes, hypertension, or lower left ventricular ejection fraction (LVEF) got a lower level of CR motivation more easily [23]. Previous studies also showed that patients suffering from limited functional abilities, sarcopenia, muscle loss, or bone loss are limited in attendance of CR [17]. Up to 16 individual health deficits, such as serum LDL, triglycerides, and BMI, were associated with low CR completion, which could be easily observed in highly frail patients [30, 31].

Thorup et al. and Brocki et al. [32, 33] designed their cardiac telerehabilitation program to solve the problem of low adherence. However, elderly patients did not fully benefit from telerehabilitation. One of the primary reasons was that they were unwilling to participate in telerehabilitation; some even felt anxious about telerehabilitation because of their unfamiliarity with IT [34]. Another non-ignorable reason is the lack of caregivers. Patients undergoing TAVR face these situations more or less, resulting in a comparatively low adherence rate of CR among those patients. As a result, it’s necessary to overcome the difficulties and solve the problem of low adherence.

## 3. CR benefits for patients undergoing TAVR

The evolution of CR for post-TAVR patients did not go well due to these patients’ poor functional status and the lack of standardised, systematic guidelines. However, a few studies still explored the applicable management and rehabilitation for post-TAVR patients. More and more evidence has proved that post-TAVR patients gained significant benefits from CR, although each study had a different program and management details (Supplementary Table 1).

### 3.1 Improvement of exercise capacity

Most studies about CR for post-TAVR patients show that patients can significantly improve their exercise capacity. Oz et al. [3] showed that the improvement of 6MWT (235.88 ± 69.36 m to 292.12 ± 54.92 m) had a moderate clinically relevant effect size [0.593, 95%CI: (0.42, 0.76); *P*=0.00]. Other indices (SPPB, 5MWT, HGS, exercise intensity, et al.) also improved after CR. Besides, at mid-term, even long-term follow-up, significant improvement of 6MWT was also confirmed in some studies [35, 36]. In a nutshell, CR had a remarkable and long-term impact on exercise capacity among patients with TAVR.

### 3.2 Improvement of cardiopulmonary function

Several studies showed the improvement of cardiopulmonary function among these kinds of patients through CR. In the study by Pressler et al. [37, 38], cardiopulmonary function was notably enhanced in patients who received CR after TAVR, as opposed to those who did not. [VO_2_ peak: 3.7 mL/min/kg, 95%CI:(1.1, 6.3), *P*=0.007; VO_2_AT: 3.2 mL/min/kg, 95%CI: (1.6, 4.9), *P*<0.001; Cardiopulmonary exercise testing (CPET) duration: 49 s, 95%CI: (6, 91), *P*=0.025]. What’s more, the improvement of cardiopulmonary function was sustained for a long period [VO_2_AT: 2.7 ml/min/kg (0.8, 4.6), *P*=0.008].

### 3.3 Improvement of psychological fitness

The positive outcomes of psychological fitness of post-TAVR patients undergoing CR seem to need more evidence compared to post-SAVR patients, although improvements can also be seen. In the study by Imran et al. [39], depression score, positive affect, negative affect and mental composite score improved significantly among post-TAVR patients (4.6 ± 3.6 to 2.3 ± 2.0, *P*< 0.01; 32.1 ± 5.8 to 35.7 ± 5.0, *P* =0.01; 15.3 ± 4.0 to 14.0 ± 4.1, *P* =0.05; 49.4 ± 9.3 to 57.6 ± 5.4, *P* < 0.01), while anxiety score not (3.0 ± 3.8 to 2.3 ± 3.2, *P* = 0.25). However, anxiety scores improved significantly in the study by Eichler et al. (5.2±4.0 to 4.0±3.6, *P* <0.001) [40]. Due to the different mental management methods in each study, there were differences in psychological fitness outcomes, but there was no doubt that CR improved psychological fitness. In light of this, suitable and optimal mental management is essential to enhance psychological wellness, and clinical physicians should keep an eye on these mental problems for patients undergoing TAVR.

### 3.4 Improvement of quality of life (QOL)

For patients undergoing TAVR, QOL is the most intuitive benefit they gain from CR. In the study of Eichler et al., the PCS (physical component scales) and MCS (mental component scales) of SF-12 increased from 35.9 ± 8.8 to 38.3 ± 8.3 (*P*=0.001) and from 47.3 ± 10.6 to 50.7 ± 10.0 (*P*=0.003) [40]. Moreover, post-TAVR patients significantly reduced the prevalence of cognitive impairment, malnutrition, and frailty (21%, 40%, 57%; *P*=0.002, *P*<0.001, *P*<0.001) through the CR program based on comprehensive geriatric assessment [21]. In general, patients improved functional independence, cognitive competence, fall risk, activities in daily living, comorbidities, and nutrition through the systematic CR program.

## 4. Post-TAVR CR evaluation

The assessment of the post-TAVR patient prior to CR consists of exercise capacity, psychological health, nutritional status, and quality of life (Table 2).

**Table 2:** TAVR CR Evaluation.

Part	Theme	Questionnaires		Items	Advantage	Score range	Other forms
Psychological health	Depression	GDS-30		30	high psychometric properties for detecting depression among older cardiac patients and screening depression change	absent depression (0-10) mild depression (11-15) moderate depression (16-20) severe depression (21-30)	GDS-15
		**PHQ-9**		9	shorter, more stable, and easier to use, and provide a direct assessment of depression symptoms for older patients	no depression (0-4) mild depression (5-9) moderate depression (10-14) moderate-severe depression (15-19) severe depression (20-27)	
		HADS	HADS-D	7	focuses more on the emotional aspects of anxiety and depression	no depression (0-7) depression disorders (8-10) severe depression (11-21)	
	Anxiety		HADS-A	7		no anxiety (0-7) anxiety disorders (8-10) severe anxiety (11-21)	
		**GAD-7**		7	good enough to screen for any anxiety disorder	minimal anxiety (0-4) mild anxiety (5-9) moderate anxiety (10-14) severe anxiety (14-20)	
Nutritional status		MNA		18	high sensitivity and specificity	malnourished (0-17) at risk of malnutrition (17-23.5) normal nutritional status (24-30)	MNA-SF
		**MUST**		3	closely associated with the change of body weight	Low Risk (0) Medium Risk (1) High Risk (2-6)	
		**RYP-heart**		24	focuses on lifestyle factors	the least healthful (24-40) moderately healthful (41-57) the most healthful (58-72)	
Quality of life	Behavior states	KI		6	assesses basic ADL function among older adults	0 (very dependent)-6 (independent)	
		**BI**		10	well-accepted, easy to use, and can be repeated periodically	0 (very dependent)-100 (independent)	
		**FIM**		18	one of the most widely used assessment measures in the rehabilitation of neurological disorders	18 (very dependent)-126 (independent)	
		**NEADL**		22	recommended for IADL in geriatric medicine	0 (very dependent)-22 (independent)	
	Health status	SF-36		36	the most widely used scale to measure HRQL	0 (the worst)-100 (the best)	SF-12
		**EQ-5D**		5	very easy to use	0 (the worst)-100 (the best)	
		**PROMIS Global 10**		10	used in the general population and individuals with chronic conditions	0 (the worst)-20 (the best)	CAT
	Frailty	EFS		8	measure multiple domains	no frailty (0-5) vulnerable frailty (6-7) mildly frailty (8-9) moderately frailty (10-11) severely frailty (12-17)	
		**FRIED Frailty score**		5	objectively measure physical functioning	non-frail (0) pre-frail (1-2) frail (3-5)	

TAVR: Transcatheter aortic valve replacement; CR: cardiac rehabilitation; GDS: Geriatric Depression Scale; PHQ-9: Patient Health Questionnaire-9; HADS: Hospital Anxiety and Depression Scores; GAD-7: General Anxiety Disorder-7; MNA: Mini Nutritional Assessment; MUST: Malnutrition Universal Screening Tool; RYP: Rate Your Plate; KI: Katz index; ADL: Activities of Daily Living; BI: Barthel Index; FIM: Functional independence measure; NEADL: Nottingham Extended Activities of Daily Living; IADL: Instrumental Activities of Daily Living; SF-36: 36-Item Short-Form Health Survey; SF-12: 12-Item Short-Form Health Survey; HRQL: health-related quality of life; EQ-5D: EuroQol five dimension scale; PROMIS: Patient-Reported Outcomes Measurement Information System; CAT: computerized adaptive test; EFS: Edmonton Frailty score

### 4.1 Exercise capacity

#### 4.1.1 Endurance exercise: CPET & 6MWT & Duke Activity Status Index (DASI)

CPET is a standard assessment at CR admission and discharge for patients with cardiovascular disease, which provides valuable information about the aerobic capacity and safety of patients in order to develop personalised exercise prescriptions. However, due to limited physical fitness and cardiopulmonary function, the tolerance of CPET still needs more consideration. Patients who attended different CR programs after TAVR presented with different attendances.

Compared with CPET, 6MWT is a simple and safe test widely used in physical function assessment during CR [41, 42]. 6MWT can be used as a preliminary test to resolve patients who were unable to perform CPET. Although 6MWT is a low-load and low-stress test, there are still a few patients who are unable to tolerate 6MWT. As a result, CR staff specially designed an exercise program for patients who were unable to perform 6MWT or those who presented disability or reduction of functional capacity [43]. Beyond that, there seems to be an alternative-Duke Activity Status Index (DASI).

DASI, a 12-item self-administered standardised scale, correlates with gold-standard measures of functional capacity validated with VO_2_ peak. DASI has validity and reliability as a measure of functional capacity in patients with CAD and HF [44, 45]. DASI provides valuable information on disease severity, effects of rehabilitation, and prognosis in patients with cardiovascular disease, has a relatively good ability for risk stratification, and shows a significant association with VO_2_ peak by CPET and 6MWT [46]. DASI estimates VO_2_ peak to measure functional capacity, which is quite suitable for post-TAVR patients, especially those unable to finish CPET and 6MWT.

#### 4.1.2 Resistance exercise: 1 repetition maximum test (1-RM) & hand grip strength (HGS) test

Not only endurance exercise, but muscular strength also maintains functional independence and reduces disease-related damage among older patients. As a result, it is also necessary to evaluate the resistance capacity at CR admission. Recommended by *Guidelines for Cardiac Rehabilitation Programs Sixth Edition*, 1-RM is a primary method to assess muscle strength and provide a reference for the resistance exercise of the post-TAVR CR program [47, 48].

Tested by a Hand Dynamometer, HGS is also a reliable and responsive measure that can be used to evaluate the resistance capacity in CR [49]. It has been shown that HGS can indicate global functional decline in older adults and as a predictor of exercise capacity when patients can’t tolerate 6MWT [50, 51].

#### 4.1.3 Balance exercise: short physical performance battery (SPPB) & 5-meter walk test (5MWT) & sit-to-stand (STS) test & timed up and go (TUG) test

SPPB is an efficient, effective, and accurate test designed to measure functional status and physical performance and classify physical limitations and frailty, including three domains of lower extremity function: the ability to stand for up to10 seconds with feet positioned in three ways, time to complete a 3-meter or 4-meter walk (quite similar with 5MWT), and time to rise from a chair for five times (STS test) [52, 53]. Focused on lower limb muscle strength and balance control, SPPB is easy to perform, with less time and materials required for the test [53]. Besides, compared with a separate 5MWT or STS test, SPPB is more comprehensive. However, it is worth noting that even though SPPB has been used for many years in miscellaneous populations and advocated for geriatric assessments, there is still relatively little experience using it and applying it to CR [52, 54]. TUG is a relatively simple screening tool used to evaluate dynamic balance ability, and it only requires a few minutes. There was a significant correlation between the TUG time and the 6MWT distance [55]. So, TUG may be able to act as a supplementary test, especially when patients are unable to perform 6MWT.

### 4.2 Psychological health: Hospital Anxiety and Depression Scores (HADS) & Geriatric Depression Scale (GDS) & Patient Health Questionnaire-9 (PHQ-9) & General Anxiety Disorder-7 (GAD-7)

Considering the clinical characteristics of TAVR patients, CR staff are more in need of a susceptible and comprehensive tool with high positive predictive values to assess anxiety and depression at post-TAVR admission [56], although it may increase false positive rates. Because it is worth giving enough psychological intervention to prevent anxiety and depression. HADS total score is sensitive and specific for detecting anxiety disorders, depressive disorders, and any mental disorder among patients with long-term conditions and is often used in geriatric rehabilitation [57]. When compared with PHQ-9 and GAD-7, HADS focuses more on the emotional aspects of anxiety and depression, excludes somatic symptoms related to emotional and physical disorders, and may avoid confounding the diagnosis in patients who have both emotional and physical illnesses, which improves the sensitivity in screening for depression and anxiety [58]. In the study of Shahab et al. [59], GDS-15, which was superior to other depression measures in older patients with depression, was sensitive to the detection of depression and helpful in identifying depressive symptoms in geriatric rehabilitation. Gonzalez-Roz et al. also found that GDS-15 best-monitored changes in depression among older patients (≥ 63 years of age) [56]. However, GDS has some obvious negative points in showing low reliability when retesting and low accuracy when testing patients with neurocognitive disorders [60]. As a depression screening tool, PHQ-9 is not only as reliable and effective as GDS-15 but also shorter, more stable, and easier to use, and provides a direct assessment of depression symptoms for older patients [61, 62]. When assessing depression, GDS-30/GDS-15 may be the first choice. GDS-30/GDS-15 shows high psychometric properties for detecting depression among older cardiac patients and screening for changes in depression during the whole post-TAVR CR program [56, 59]. HADS-D or PHQ-9 is a convenient and alternative tool to screen depression for TAVR patients with cognitive disorders or low adherence. In terms of evaluating anxiety, GAD-7 is good enough to screen for any anxiety disorder [63], but HADS is designed for overall anxiety symptoms among patients from medical clinics, not for patients with specific anxiety symptoms or the general population [64]. As a result, HADS-A may be a better choice because it is more suitable for the clinical characteristics of TAVR patients. Further studies are needed to determine whether GAD-7 is reliable for post-TAVR patients.

### 4.3 Nutritional status: Mini Nutritional Assessment (MNA) & Malnutrition Universal Screening Tool (MUST) & Rate Your Plate (RYP) survey

In the study of Katano et al. [65], approximately 50% of patients undergoing Phase Ⅱ CR were malnourished or at risk of malnutrition. MNA could be used to assess nutritional status, and so could the MNA-SF. While in the study of Katano et al. [66], MNA-SF was also the predictor of the extent of functional recovery for older HF patients after CR. MUST, closely associated with the change of body weight, takes less time but has lower sensitivity when compared with MNA [67]. So, MUST is more suitable for those who adhere poorly to the CR. RYP is a dietary questionnaire for the assessment of cholesterol screening and education, and has been updated into RYP-heart [68]. RYP-Heart is a quick, inexpensive tool biased towards cardiovascular risks and is likely sensitive among older patients, but the utility of RYP needs further evaluation during CR [68, 69]. For post-TAVR patients undergoing CR, a comprehensive and detailed nutritional status assessment seems necessary. Although MNA takes a longer time, up to about 15 minutes, not all the questions are self-reported, and some can be answered by guardians. In summary, MNA may be the first choice when assessing nutritional status. If patients show low adherence, MUST or MNA-SF can be used as a quick alternative screening tool.

### 4.4 Quality of life

#### 4.4.1 Behaviour states

Katz index (KI) & Barthel index (BI) & Functional Independence Measure (FIM) of Basic Activities of Daily Living (BADL) and Nottingham Activities of Daily Living (NADL) of Instrumental Activities of Daily Living (IADL)

For older patients, ADL is divided into BADL and IADL. KI and BI are conventional methods to assess basic ADL function among older adults [70], which makes them an appropriate choice in post-TAVR CR [71, 72]. KI is not sensitive enough to detect a small degree of variation in impairment, while BI may not be reliable enough if the changes in BADL are extreme. However, compared with KI, BI is well-accepted, easy to use, and can be repeated periodically [72]. Designed to provide an improvement of BI at first [73], FIM is one of the most widely used assessment measures in the rehabilitation of neurological disorders, and the 7-point Likert scale is used in scoring, which reports the difference between maximum independence and maximum dependence of individuals with more caution and accuracy. However, it takes too much time to complete, which may influence adherence of post-TAVR patients [73]. NEADL was developed to assess the IADL of patients with stroke living at home at first and has proved good validity, reliability, and responsiveness in stroke, hip replacement, pulmonary disease, and multiple sclerosis [74]. In geriatric medicine, NEADL is recommended for IADL, which is less applicable to highly dependent patients [71]. Among the CR programs for post-TAVR patients, NEADL was scarcely used to assess their functionality. The IADL test was only used in a previous study to act as a part of the customised Frailty Index [40]. It has been speculated that among TAVR patients, many of them may be highly dependent with advancing age and disability. They have difficulty completing BADL tests, but still fewer IADL tests. In the early stages, CR staff are supposed to pay more attention to improving post-TAVR patients’ BADL skills. A comprehensive IADL test is not indispensable unless post-TAVR patients show good functional capacity or have finished a period of the CR program and sought further improvement.

#### 4.4.2 Health status

EuroQol Questionnaire (EQ-5D) & 36-Item Short-Form Health Survey (SF-36) & PROMIS Global 10

In the study of Haywood et al. [75], they advised that EQ-5D is recommended when a more succinct health status assessment is required, particularly for patients who may experience a substantial change in health. In contrast, SF-36 is recommended when a broad-ranging and detailed evaluation of health-related quality of life (HRQL) is needed, particularly for older people with lower levels of morbidity. However, although EQ-5D is very easy to use, it has a prominent problem with ceiling effects [76]. As a result, SF-36 is the most widely used scale to measure HRQL and is widely accepted for evaluation among older patients, which is sensitive to change [75, 77]. SF-36 and SF-12 are intended to measure “general healing concepts not specific to any age, disease, or treatment group”, and their PCS and MCS scores show high consistency, respectively [78, 79]. Developed from the Patient-Reported Outcomes Measurement Information System (PROMIS) and adapted from measures such as SF-36 and EQ-5D [77], PROMIS Global 10 is used in the general population and individuals with chronic conditions [80]. On top of the PROMIS Global 10, the PROMIS computerised adaptive test (CAT) is a sophisticated computer assessment tool to assess a patient’s symptomatology precisely [81]. Compared with traditional fixed PROMIS, CAT is more applicable to the patients, resulting in questions that are likely more relevant to that patient. Moreover, fewer CAT questions will be required to obtain similar or even more precise measurements [82]. In post-TAVR CR assessment, a broad-ranging assessment of HRQL is much more required. Currently, the SF-36 questionnaire is the most widely used quality-of-life instrument in the world, and PROMIS instruments are gaining popularity as well [83]. However, the evidence of PROMIS application in CR is lacking, so SF-36 is a better choice.

#### 4.4.3 Frailty

Edmonton Frailty score (EFS) & FRIED Frailty score

FRIED had a higher sensitivity and false-positive rate, while EFS had better specificity and false-negative rate. FRIED objectively measured physical functioning but did not consider other domains, including cognition. EFS, on the other hand, measured multiple domains. However, it is worth noting that both require significant time to perform, which may not be suitable for CR assessment [84].

### 4.5 Cognitive function: Mini-Mental State Examination (MMSE)

MMSE consists of orientation, memory, attention and concentration, language, and spatial ability, and it is the most recognised cognitive function test among healthcare professionals [85]. MMSE score is a predictor of 6MWT, which, to some extent, decides exercise intervention for patients undergoing TAVR at admission. Likewise, it is supposed to be used with a BADL test such as BI because cognition is strongly linked with functionality [72]. Overall, MMSE scores may determine the degree of intervention, attention, and supervision of post-TAVR patients during the CR program.

## 5. Post-TAVR CR program

### 5.1 Introduction and description

Developed from *Consensus of Chinese Experts on Exercise Rehabilitation after Transcatheter Aortic Valve Replacement*, *Guidelines for Cardiac Rehabilitation Programs Sixth Edition, Chinese Expert Consensus on the Application of Clinical Norms of Six-minute Walk Test*, and researches summarized from Supplementary Table 1, Post-TAVR CR program has the characteristics of a shorter time, a higher efficiency, a less cost and is actionable and adaptive in all CR centers and for all CR staff [86-88]. It is worth mentioning that most researchers focused on the exercise evaluation and intervention, especially on aerobic exercise for post-TAVR patients. However, post-TAVR CR is supposed to be a multidimensional and comprehensive program with structured personalisation and multidomain assessment, which means other kinds of evaluations and interventions deserve equal emphasis. Post-TAVR CR evaluation is structured into 5 main parts: cognitive function screening test, exercise function test, psychological health evaluation, nutritional status evaluation, and quality of life evaluation. Accordingly, post-TAVR CR intervention consists of 4 main parts: physical intervention, psychological intervention, nutritional intervention, and life management. CR staff are supposed to evaluate the functional capacity briefly and develop a personalised program for post-TAVR patients (Fig. 1).

**Figure 1.**
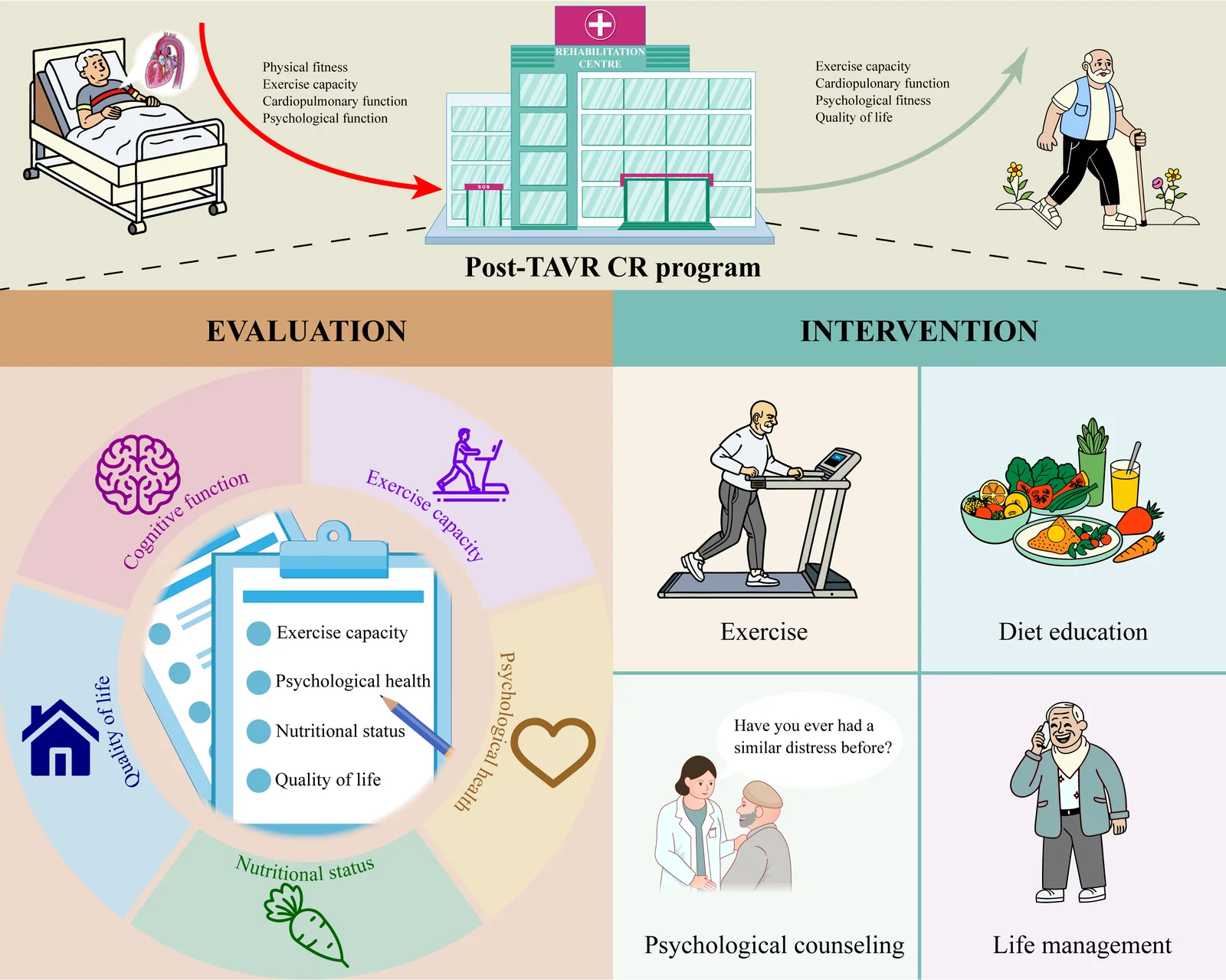
Post-TAVR CR program.

### 5.2 Step 1: Cognitive function screening test

The cognitive function underlies the progress of the CR program. MMSE is recommended to evaluate cognitive function at post-TAVR admission [21, 40, 43]. Whatever the degree of cognitive function, a post-TAVR patient is supposed to participate in the post-TAVR CR program. If a patient scores above 23, he/she is able to participate in the post-TAVR CR program smoothly. If a patient scores less than or equal to 23 but greater than or equal to 5, he/she should participate in post-TAVR CR, but CR staff should also evaluate his/her cognitive function regularly and supply individualised cognitive education and training. If a patient scores below 5, he/she shows high cognitive impairment at the present stage. Besides cognitive function evaluation and training, he/she is suggested to undergo the post-TAVR CR program for as much time as possible. CR staff should also pay more attention to attendance than completion for these patients.

### 5.3 Step 2: Exercise function test & Psychological health evaluation & Nutritional status evaluation & Quality of life evaluation (Regardless of order)

Exercise function tests mainly consist of endurance exercise tests and resistance exercise tests. At first, CR staff must use the AACVPR category to evaluate the risk of exercise [86]. It is recommended that all post-TAVR patients perform CPET to evaluate endurance function as much as possible if they do not meet the contraindications for CPET [86]. Otherwise, he/she is supposed to perform 6MWT as an alternative test. DASI is an alternative to roughly evaluate a patient’s exercise function if the patient is not even able to tolerate 6MWT. When testing a patient’s resistance exercise function, a 1-RM evaluation is preferable.

Considering the universality and high efficiency, it is better to use GDS-30/GDS-15 and HAD-A, MNA, and SF-36 to evaluate psychological health, nutritional status, and quality of life, respectively. Most post-TAVR patients easily accept them and take only a few minutes to complete (Fig. 2).

**Figure 2.**
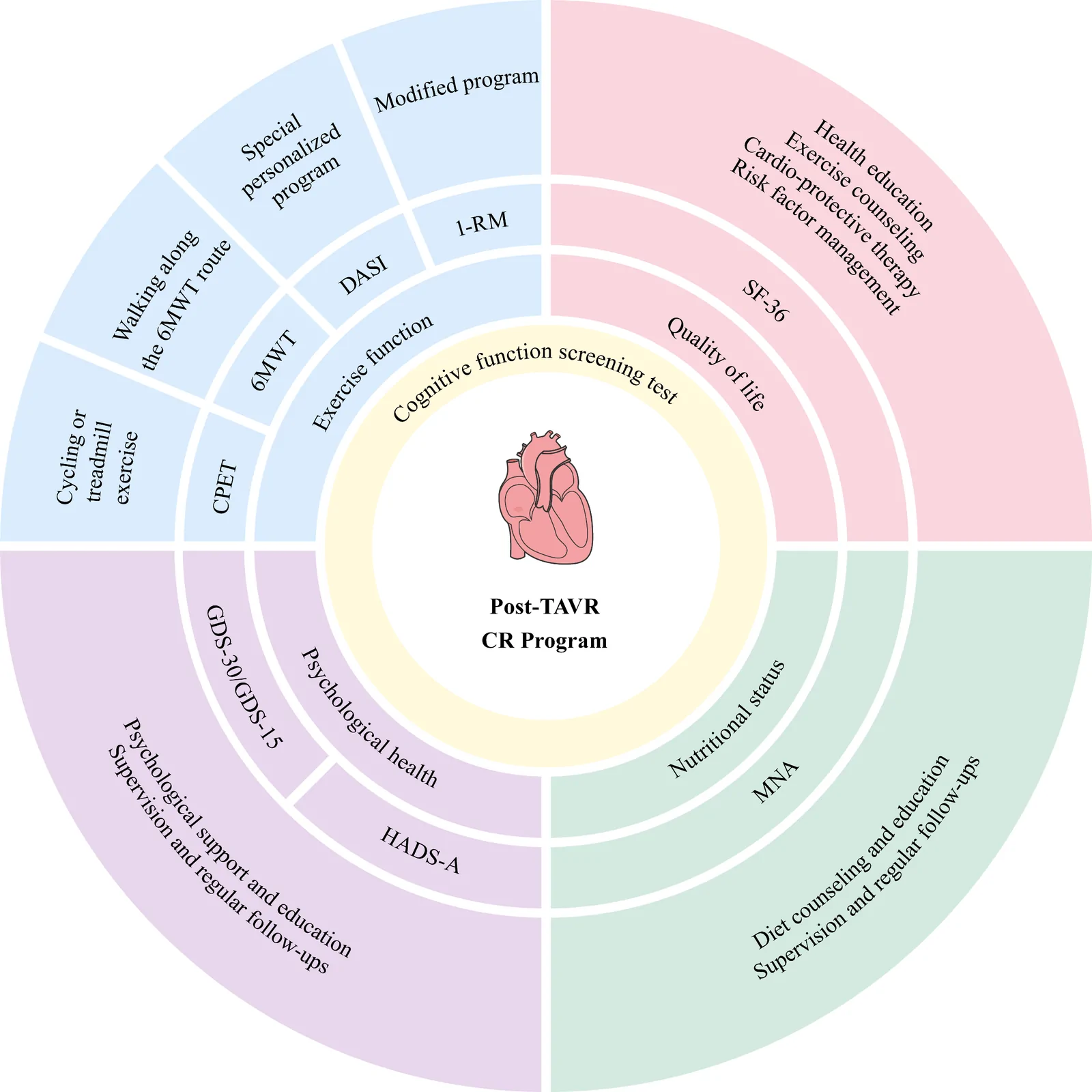
Construction of a post-TAVR CR program.

### 5.4 Step 3: Develop a post-TAVR CR program

#### 5.4.1 Aerobic exercise

Considering the complicated physical conditions of post-TAVR CR patients, aerobic exercise is the basic and core program of post-TAVR CR. All the post-TAVR patients who tolerate CPET are supposed to undergo a training program of cycling or treadmill exercise for 30 min/day and 6 days/week [3]. The expected duration is 12 weeks, divided into 4 phases, each lasting 3 weeks [3]. Summarising these CR programs, it is discovered that the intensity lacks a consolidated standard. However, CR staff have enough objective indices to set an appropriate target for post-TAVR CR patients. Considering the low physical function at the beginning, the warm-up phase is vital, with the intensity starting at 0W, gradually increasing to 100% of the target intensity and lasting for 5-10 minutes [89, 90]. The target intensity is referred from those alternative objective indexes: 40%-70% VO_2_ peak, 60%-85% of maximal HR, 30-50% maximal watt or anaerobic HR±5 [36, 37, 39, 89]. As a patient adapts to the intensity referred from the objective indexes, subjective intensity occupies a more significant place in guiding an exercise program. Borg score of 10-14 is recommended as the target intensity [91, 92]. The specific intensity can be adjusted according to the risk of exercise evaluated by the AACVPR category. The cool-down phase should last 3 minutes with a gradual load reduction. A patient’s physical function is supposed to be reevaluated every 3 weeks, and the CR staff reset the target intensity according to the result.

**Table 3:** Absolute and Relative Contraindications to Post-TAVR Exercise Test

Absolute Contraindications
•Acute aortic dissection
•Acute infectious disease that could interfere with or be exacerbated by exercise
•Acute liver and kidney failure
•Acute myocarditis, pericarditis, or endocarditis
•Acute myocardial infarction, within 2 days
•Acute pulmonary embolism, pulmonary infarction, or deep vein thrombosis
•Acute respiratory failure
•Decompensated heart failure
•Ongoing unstable angina
•Physical disability that excludes the patients from daily exercise
•Severe coarctation of the aorta, or descending aortic aneurysm
•Symptomatic cardiac arrhythmia with hemodynamic compromise
•Symptomatic severe aortic stenosis
•Uncontrolled asthma
**Relative Contraindications**
•Atrial thrombus
Bradyarrhythmia, or high-degree atrioventricular blocks
•Hypertrophic obstructive cardiomyopathy with severe resting gradient (>25mmHg)
•Known obstructive >50% left main coronary artery stenosis or occlusion
•Mental impairment limiting participation
Moderate to severe aortic stenosis without obvious symptoms
•Peripheral SpO2 <85% when resting
•Recent stroke or transient ischemic attack
•Tachyarrhythmia with uncontrolled ventricular rates
Uncorrected medical conditions, such as hemoglobin< 9 g/dL, important electrolyte imbalance, and hyperthyroidism
•Untreated or uncontrolled hypertension (SBP>180 mmHg and/or DBP>110 mmHg)

TAVR: transcatheter aortic valve replacement; SBP: systolic blood pressure; DBP: diastolic blood pressure

If a patient is not able to tolerate CPET, walking along the 6MWT route is recommended according to Genta et al.’s program [92]. Patients are supposed to undergo a daily session of up to 30 min. During the warm-up phase, the walking speed is supposed to gradually increase to 100% target intensity in 5-10 minutes. The target intensity is supposed to be a 70% to 80% average walking speed of 6MWT and BORG 14-17 if a patient is at a low risk of the AACVPR category. Otherwise, it is supposed to be 50%-60% average walking speed and BORG 11-13 [88]. The cool-down phase should last 3 minutes with a gradual load reduction. Another noteworthy point is that the program lasts only 6 weeks to make the CR staff reevaluate the patients’ physical function and help them undergo cycling or treadmill exercise as early as possible.

If a patient cannot tolerate 6MWT at admission, he/she is supposed to undergo a special personalised program training, according to Genta et al.’s research [90]. Likewise, patients who are not allowed to perform any exercise test are still recommended to undergo the program despite insufficient evidence (Table 3). The training is followed until a patient is able to tolerate CPET or 6MWT. The target intensity could refer to DASI.

#### 5.4.2 Resistance exercise& Balance exercise

Different from the vital place of the CR program, resistance exercise aims to be an advanced exercise. According to Eichler et al.’s research, a post-TAVR patient is allowed to undergo resistance exercise if his/her workload is greater than 1.0W/kg [40]. As for the resistance exercise program, Tamulevičiūtė-Prascienė et al.’s exercise program is recommended (Table 4) [36]. It was performed 3 times per week for 20-25 minutes. The expected duration is 12 weeks, divided into 4 phases, each lasting 3 weeks. During the first week, each resistance exercise is supposed to be done in 3 sets with 5-10 repetitions, with a 30-second rest between sets and a 3-minute rest between different resistance exercises. The target intensity is supposed to be BORG 11 and < 30% 1-RM. During the second and third week, the exercise volume increases to 15 repetitions in each set. The target intensity is supposed to be BORG 12-13, 30-50% 1-RM in week 2, and BORG ≤15, 50-60% 1-RM in week 3.

Apart from resistance exercise, balance exercise helps advance frailty. A post-TAVR patient is supposed to undergo balance exercise 3 times per week, lasting 12 weeks. From easy/known to harder/unknown, each balance exercise is supposed to be done for 2 minutes, with a 1-minute rest or stretching between different balance exercises. Closing one's eyes is allowed to increase difficulty. All the post-TAVR CR patients are encouraged to undergo balance exercises with SPPB as a monitor to screen for change in balance function.

**Table 4:** Modified Resistance and Balance exercise program.

Type	Time	Training Program	Routine	Intensity	Duration	Frequency
Resistance Exercise	Week 1	Knee extension with a resistance band while sitting, changing legs (90° flexion in knee) Legs abduction with a resistance band while sitting (90° flexion in the knee) Lifting heels to tiptoe with small weights on ankles while standing Lifting legs and putting them down while sitting with a small weight on each ankle (90° flexion in the knee)	3 sets with 5-10 repetitions 30 seconds rest between sets 3 min rest after each exercise	BORG 11 and < 30% 1-RM	20-25 minutes	3 times/week
	**Week 2**	Leg press Abduction of the thigh with weight on the ankle while standing Lifting heels to tiptoe with small weights on ankles while standing Stepping up and down a stair with weights on ankles	3 sets with 15 repetitions 30 seconds rest between sets 3 min rest after each exercise	BORG 12-13 and 30-50% 1-RM	20-25 minutes	3 times/week
	**Week 3**	Leg press Abduction of the thigh with weight on the ankle while standing Adduction of the thigh with weight on the ankle while standing Squats with sitting on a chair with/without a hand on the back of a chair	3 sets with 15 repetitions 30 seconds rest between sets 3 min rest after each exercise	BORG ≤15 and 50-60% 1-RM	20-25 minutes	3 times/week
Balance Exercise	Week 1	Sitting with a foot on an uneven surface Standing on an uneven surface with one foot, changing feet Standing with one foot in front of the other Putting the weight from heel to toe and back Walking along a line forward/backward, putting one foot in front of the other	2 min of exercise 1 min rest /stretching after each exercise	—	15 minutes	3 times/week
	**Week 2**	Standing with one foot in front of the other on an uneven surface Switching feet position on an uneven surface Standing on one foot with/without a hand on the back of a chair Walking along a line forward/backward, putting one foot in front of the other	2 min of exercise 1 min rest /stretching after each exercise	—	15 minutes	3 times/week
	**Week 3**	Standing with one foot in front of the other on an uneven surface Switching feet position on an uneven surface Catching a ball while standing on an uneven surface Touching the ground with tiptoes to the different side	2 min of exercise 1 min rest /stretching after each exercise	—	15 minutes	3 times/week

As shown in Table 4, it is the most detailed and reasonable exercise program among post-TAVR patients up to now. However, the study was an open-label and single-center trial with a heterogeneous patient population, significant dropouts, and missing data. The benefit and efficacy of the program need more evidence to be confirmed, and more correlational studies are warranted to establish the model of resistance and balance exercise for post-TAVR patients.

#### 4.4.3 Nutritional intervention & psychological intervention

Among post-TAVR CR programs, the mainstay of nutritional intervention is diet counselling and education. It is noteworthy that nutritional intervention is supposed to be completed by both CR staff and dietitians. So is the psychological intervention. The mainstay of psychological intervention is psychological support and education. In the program of Völler et al., several viable alternatives, such as stress management, Tai Chi, and progressive muscle relaxation, were presented [40]. In conclusion, as for CR staff, their primary task is evaluation, supervision, education, and feedback. During the whole post-TAVR CR program, it is better to find psychological and nutritional diseases promptly and refer them to outpatient clinics to solve them under the direction of professionals. Moreover, it is also vital for CR staff to monitor patients' nutritional and psychological conditions, communicate positively with dietitians and psychologists, and promptly adjust the intervention with the help of professionals (Fig. 3).

**Figure 3.**
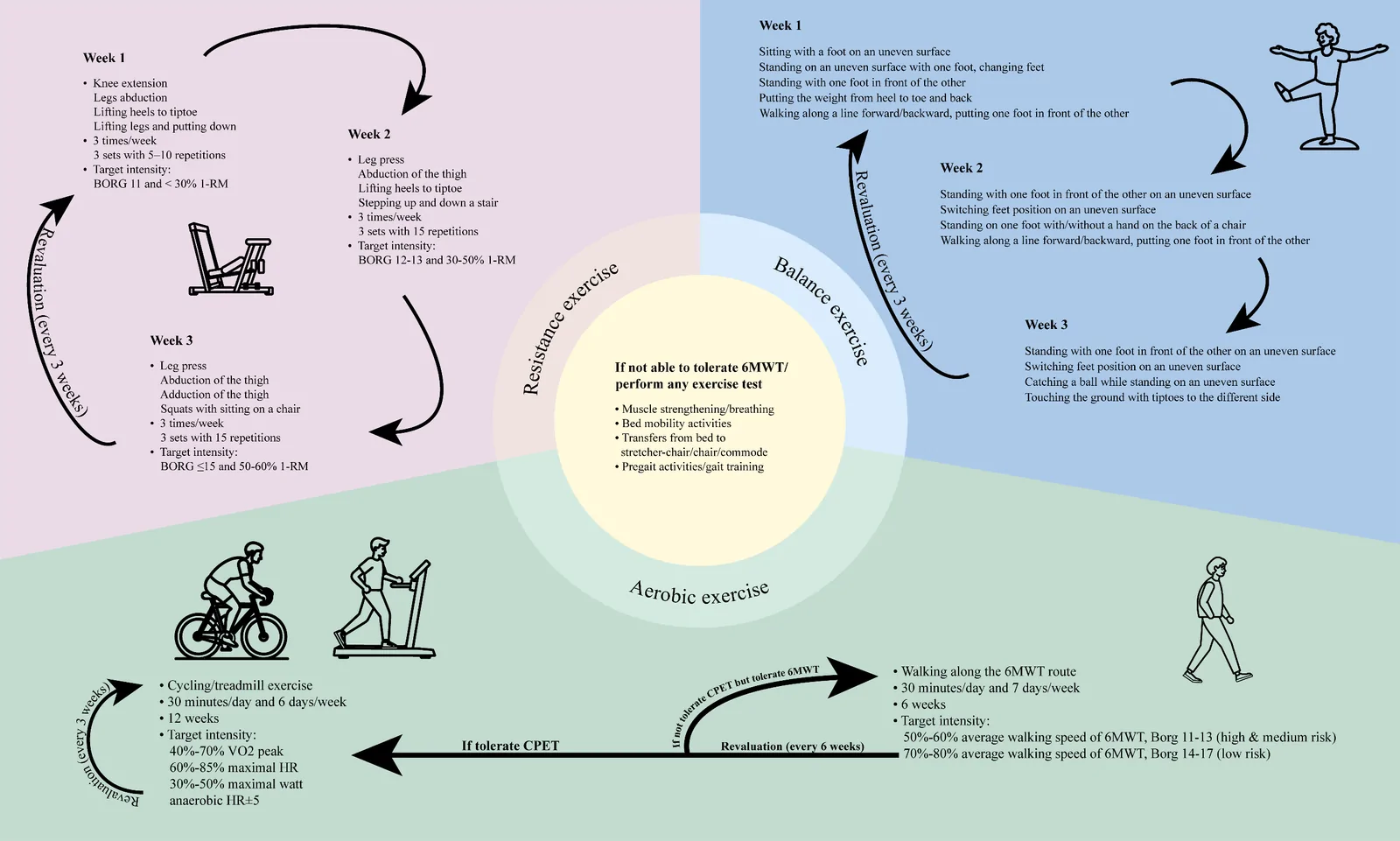
Exercise interventions of the post-TAVR CR program.

#### 4.4.4 Life Management

Life management is a comprehensive, individualised process, and the goal of life management is to reduce risk factors and improve the quality of life as recommended by *Guidelines for Cardiac Rehabilitation Programs Sixth Edition*. Among these programs, health education, exercise counselling, cardio-protective therapies, and risk factor management are common interventions besides exercise, psychological and nutritional intervention [36, 39, 40, 92-94]. Life management is a long period of overwhelming change, which needs close cooperation and joint effort among CR staff, post-TAVR patients, and specialists.

#### 4.4.5 Difficulties and barriers still faced

The post-TAVR CR program, despite having a primary program developed, still faces institutional and systemic barriers. Firstly, policy support is the key to promoting the post-TAVR CR program, which makes post-TAVR patients highly prioritise rehabilitation and maintain a strong sense of enthusiasm. Secondly, urban vs. rural disparities significantly influence the adherence of post-TAVR CR patients. For patients from urban areas, it is too hard to keep a high frequency of going to the rehabilitation center, except on the evaluation day. As a result, the telerehabilitation program is supposed to be widely used to guarantee rehabilitation quality. Thirdly, CR staff training and regional CR networks are quite important. Besides general CR training, CR staff are supposed to provide customised service based on the characteristics of different regions. It is not only beneficial to patient management but also provides social support.

## 6. Conclusion

It is obvious that the rehabilitation program provides benefits to post-TAVR patients, even though it is only in its primary form. Our study developed a relatively reasonable and standardised post-TAVR CR program based on “The Five Prescriptions”. Nevertheless, further studies are needed that provide compelling evidence, particularly those that involve substantial sample sizes and multicenter research. The Ultimate goal of the rehabilitation program is to implement individualisation and standardisation.

## Supplementary Materials

The Supplementary data can be found online at: www.aginganddisease.org/EN/10.14336/AD.2025.0716.
