# Supplementary material for: Optimal Evaluation and Treatment of Cardiac Rehabilitation for Patients Following TAVR: A Personalized Program Based on The Five Prescriptions
Source: Aging Dis. 2026 Oct 4;17(6):3160–76. doi: 10.14336/AD.2025.0716 (PMC13653704; doi:10.14336/AD.2025.0716)
Supplement: Supplementary file 1 [file AD-17-6-3160-s.pdf]

SUPPLEMENTARY DATA

Optimal Evaluation and Treatment of Cardiac Rehabilitation for Patients Following TAVR: A Personalized Program Based on The Five Prescriptions

Xue-Song Han, Xiao-Jia Sun, Yong Miao, Zhi-Yuan Tian, Yong-Chao Zhang, Zong-Yan Li, Gang Wang, Qiang Hu

© 2025. Han XS et al. Published online at http://www.aginganddisease.org/EN/10.14336/AD.2025.0716

SUPPLEMENTARY DATA

Supplementary Table 1.

<table>
<tr><th rowspan="2">Study</th><th colspan="4">CR program</th><th rowspan="2">Evaluation</th><th rowspan="2" colspan="2">Main findings</th></tr>
<tr><th>Physical intervention</th><th>Psychological intervention</th><th>Nutritional intervention</th><th>Life management</th></tr>
<tr><td colspan="8">Phase I</td></tr>
<tr><td rowspan="2">Fauchère et al.[1]</td><td rowspan="2">Supervised gymnastics, aerobic and respiratory training
Low/medium intensity, derived from Borg scale
2-3 times/day, 6 days/week</td><td rowspan="2">—</td><td rowspan="2">—</td><td rowspan="2">—</td><td rowspan="2">NYHA-class
Karnowski-Index
EURO score
FIM score
HADS score
6MWT</td><td>TAVR</td><td>SAVR</td></tr>
<tr><td>6MWT: 147.5±101.7 to 231.7±132.7m
FIM motor: 67.9±7.8 to 76.8±8.0
FIM cognitive: 28.0±2.8 to 29.6±3.0</td><td>6MWT: 200.6±96.2 to 283.4±117.1m
FIM motor: 68.6±11.5 to 78.7±7.6
FIM cognitive: 28.6±3.7 to 30.5±3.1
Depression score (HADS): 4.6±3.4 to 2.7±2.3</td></tr>
<tr><td rowspan="2">Kleczynski et al.[2]</td><td rowspan="2">Cardiovascular exercise and resistance exercise: cardiovascular treadmill (walk) and cycloergometer (with no resistance), functional exercise such as “sit to stand to sit”
Exercise intensity derived from Borg scale
15-30min/day, 6 days/week, 2 weeks</td><td rowspan="2">Psychosocial management: motivation and stress relief</td><td rowspan="2">—</td><td rowspan="2">—</td><td rowspan="2">5MWT
6MWT
HGS
KI of ADL
KCCQ
Echocardiography</td><td>CR</td><td>DH</td></tr>
<tr><td colspan="2">30 days:
5MWT: 5.1±0.9s vs. 5.8±1.3s
6MWT: 397±24m vs. 384±29m
HGS: 36±9.3kg vs. 28±12.1kg
KI of ADL: 5.0±0.8 vs. 4.8±1.0
KCCQ: 82.5±15.0 vs. 76.5±12.4
6 months:
6MWT: 426±34m vs. 392±19m
HGS: 38±7.3kg vs. 30±10.1kg
KI of ADL: 5.0±0.8 vs. 4.7±1.2
KCCQ: 86.5±17.0 vs. 78.5±11.4
12 months:
6MWT: 410±22m vs. 389±18m
HGS: 35±10.5 vs. 30±11.6</td></tr>
<tr><td rowspan="2">Xu et al.[3]</td><td rowspan="2">Supervised exercise training: mobilization training in bed, "sit to stand" transferring training, and aerobic walking training
Borg scale 11 to 12 scores
30min/time, 1 time/day, 3-5 times/week</td><td rowspan="2">Psychological management</td><td rowspan="2">Dietary advice</td><td rowspan="2">Disease education
Physical activity counseling</td><td rowspan="2">6MWT
NYHA-class
HGS test
STS test
Arm-curl test
mMRC scale
DASI</td><td>IMT plus usual CR</td><td>Usual CR</td></tr>
<tr><td colspan="2">6MWT (1 month and 3 months): 41.51 (1.82, 81.21)
FEV_1 (1 month): 0.21 (0.02, 0.40)
MIP (1 month): 7.76 (1.44, 14.09)
BADL (1 month): 9.27 (2.06, 16.48)</td></tr>
</table>

© 2025. Han XS et al. Published online at http://www.aginganddisease.org/EN/10.14336/AD.2025.0716

	IMT<br>Warm-up phase: breathing control training and chest expansion and stretching exercises<br>5min/time, 1 time/day, 3-5 times/week<br><br>Formal training, inspiratory muscle training:<br>Moderate to high resistance to inspiration, starting at 30% of each participant's baseline MIP increased to at least 60%<br>10min/time, 1 time/day, 3-5 times/week<br><br>Recovery phase, respiratory training:<br>Low resistance, less than 30% of MIP<br>5min/time, 1 time/day, 3-5 times/week				PSQI<br>BI<br>FS-14<br>KCCQ<br>SF-12<br>Lung Function		
						Before CR	After CR
Yu et al.[4]	1-3 days before TAVR:<br>Systematic respiration exercise<br>5-10 min/time, 2 times/day<br><br>Within 24 h after TAVR:<br>In-bed passive/active incremental exercises, respiration training, calf muscle pump function and ankle movement<br>5-10 min/time, 2 times/day<br><br>After 24 h and before discharge:<br>In-bed passive/active cycling, sitting upright, bed-chair transfer, standing and walking, low intensity of resistance training, 6MWT (when clinically feasible)<br>10-15 min/time, 2 times/day<br><br>1-month after discharge:<br>Respiration training, resistance training, walking, gymnastics, and aerobic exercise<br>10-25 min/time, 5-6 times/week.	Psychological support	Nutrition support	Patient education	6MWT<br>BI<br>MMSE<br>HADS<br>Fried scale<br>NYHA-class<br>MNA<br>METs	MMSE: 26.2±2.8 to 27.3±2.5<br>MNA: 22.8±3.1 to 25.1±2.7<br>Fried scale: 3.82±0.9 to 1.7±1.1<br>BI: 89.0±8.6 to 95.5±6.5<br>6MWT: 218.8±114.3 to 291.9±98.8m<br>METs: 2.9±0.6 to 3.4±0.5	
Phase II							
Zanettini et al.[5]	If unable to perform 6MWT and/or with dependence in basic activities:<br>Bed exercises, sitting callisthenics	Psychological support<br>Therapy with	Nutritional evaluation and intervention	—	NYHA-class<br>EQ-5D<br>BI	Before CR	After CR

© 2025. Han XS et al. Published online at http://www.aginganddisease.org/EN/10.14336/AD.2025.0716

	and ambulatory exercise<br>6 days/week<br>If mild disability and/or moderate reduction of functional capacity during the 6MWT:<br>Interval or steady-state aerobic training: Cicloergometer or treadmill<br>6 days/week<br><br>Callisthenics<br>6 days/week	antidepressants in case of persistent or severe depression			MMSE<br>GDS<br>6MWT<br>CIRS<br>MUST	6MWT: 210±87 to 275±97m<br>EQ VAS: 54±14 to 75±11<br>BI: 84±21 to 95±10	
						TAVR	SAVR
Russo et al.[6]	In the morning:<br>Respiratory session:<br>Low/medium intenisty, derived by a category ratio Borg scale<br>30min/time, 1 time/day, 6 days/week, 2 weeks<br><br>Aerobic session:<br>Cycling (an arm ergometer if not able to cycle)<br>Constant work rate, ≤70% of the maximal predicted HR<br>10-30min/time, 1 time/day, 6 days/week, 2 weeks<br>In the afternoon:<br>Callisthenic session:<br>30min/time, 1 time/day, 6 days/week, 2 weeks	—	—	—	LVEF<br>Haemoglobin values<br>NYHA-class<br>BI<br>6MWT<br>CPET	Δ6MWT: 60.4±46.4m<br>ΔBI:9.3±12.3	Δ6MWT: 72.3±57.3m<br>ΔBI:10.6±13.2
						TAVR	SAVR
Völler et al.[7]	Aerobic exercise:<br>Training on a bicycle ergometer in different workload classes, depending on the initial exercise intensity, outdoor walking and gymnastics in groups or single<br>4-5times/week, 3 weeks<br>Resistance exercise of the lower extremities<br>4-5times/week, 3 weeks	Psychological support: depended on the need and wish of the patients<br>Psychological education: stress management, Tai Chi, and progressive muscle relaxation	—	—	LVEF<br>NYHA-class<br>6MWT<br>HADS<br>Cycle-exercise test	6MWT: 28.13 (20.59, 36.15)<br>Exercise capacity (bicycle exercise test): 19.84 (4.76, 37.08)	6MWT: 45.51 (38.87, 50.38)<br>Exercise capacity (bicycle exercise test): 26.72 (21.62, 32.02)<br>Anxiety score (HADS): -23.60 (-30.09, -16.51)<br>Depression score (HADS): -23.36 (-29.11, -17.12)
						TG	UC
Pressler et al.[8]	Endurance exercise: cycle ergometers<br>Moderate intensity, starting with 20 min at 40% VO_2peak and gradually	—	—	—	NYHA-class<br>6MWT<br>KCCQ		

© 2025. Han XS et al. Published online at http://www.aginganddisease.org/EN/10.14336/AD.2025.0716

	increasing to 45 min at 70% VO_2peak by week 8<br>2 times in week 1, 3 times/week in week 2 to 8<br><br>Resistance exercise: bench press, rowing, shoulder press, pulldown, leg press<br>Starting with 1 set with 10 repetitions at intensities of 30% of the 1-RM and gradually increasing to 3 sets with 15 repetitions at 50% to 60% of 1-RM. Starting in week 2, subsequent to the endurance exercise part in 2 of the 3 weekly workouts				LV-EF<br>SF-12<br>CPET<br>Muscular strength	VO_2peak: 3.7 mL/min per kg (1.1, 6.3)<br>VO_2AT: 3.2 mL/min per kg (1.6, 4.9)<br>CPET duration: 49 s (6, 91)<br>Physical limitation (KCCQ): 19.2 (4.1, 34.2)<br>Overall summary (KCCQ): 7.3 (0.2, 14.4)<br>Clinical summary scores (KCCQ): 12.4 (3.4, 21.4)	
Eichler et al. [9]	Endurance exercise: bicycle training<br>Continuous or interval<br>30 min, 5 times/week<br><br>Additional strength training (If >1.0 W/kg): common weight machines 30–50% of one RM<br><br>If appropriate:<br>Outdoor walking, gymnastics, aqua gymnastics and spinal gymnastics in groups	Psychological support	Diet counselling	Patient education<br>Risk factor management	6MWT<br>SF-12<br>HADS<br>MMSE<br>MNA<br>ADL<br>IADL<br>TUG<br>Echocardiography<br>Symptom limited bicycle exercise test	Before CR	After CR
						Δ6MWT: 56.3±65.3m<br>Maximum exercise capacity (bicycle): 50.8±20.3W to 58.9±21.3W<br>The proportion of frail patients: 36.9% to 27.9%<br>PCS (SF-12): 35.9±8.8 to 38.3±8.3<br>MCS (SF-12): 47.3±10.6 to 50.7±10.0<br>Anxiety score (HADS): 5.2±4.0 to 4.0±3.6	
Genta et al.[10]	Aerobic session: cycling or treadmill exercise<br>Minimun workload to a value of 14 on the Borg Rating of Perceived Exertion Scale<br>30 min/time, 2 times/day (except for Saturday, only in the morning), 6 days/week, 3 weeks<br><br>If temporarily contraindicated treadmill or cycling exercise:<br>Walking along the 6MWT route: 30min/time, 1 time/day, every day<br><br>Pedal exercise:<br>0W<br>Up to 30 min/time, 2 times/day, every day<br><br>Respiratory session:<br>40min/time, 1 time/day, every day	Psychological education	Dietary education and counseling	—	Echocardiography<br>BI<br>MFS<br>6MWT<br>CIRS-CI	TAVR	SAVR
						BI: 67±24 to 85±17<br>MFS: 36±22 to 30±20<br>6MWT: 162±87 to 240±92m	BI: 79±21 to 93±14<br>MFS: 25±19 to 20±12<br>6MWT: 216±82 to 332±95m

© 2025. Han XS et al. Published online at http://www.aginganddisease.org/EN/10.14336/AD.2025.0716

	If bedridden or markedly disabled or with a high risk of falls at admission:<br>Individualized physical therapy interventions for early mobilization<br>Followed the above described training protocol when their condition improved						
						TG	UC
Pressler et al.[11]	Endurance exercise:<br>Cycle ergometers<br>Moderate intensity, starting with 20 min at 40% VO_2peak and gradually increasing to 45 min at 70% VO_2peak by week 8<br>2 times in week 1, 3 times/week in week 2 to 8<br>Resistance exercise:<br>Bench press, rowing, shoulder press, pulldown, leg press<br>Starting with 1 set with 10 repetitions at intensities of 30% of the 1-RM and gradually increasing to 3 sets with 15 repetitions at 50% to 60% of 1-RM.<br>starting in week 2, subsequent to the endurance exercise part in 2 of the 3 weekly workouts	—	—	—	NYHA-class<br>6MWT<br>KCCQ<br>LV-EF<br>SF-12<br>CPET<br>Muscular strength	VO_2AT: 2.7 ml/min/kg (0.8, 4.6)	
						CR	No CR
Butter et al.[12]	Ergometer and treadmill training, Thera Band use, strength and muscle building, swimming<br>3weeks	Psycho-social health	—	Health behavior change and education<br>Lifestyle risk factor management<br>Medical risk factor management<br>Cardio-protective therapies<br>Long-term management	LVEF<br>NYHA-class<br>NT-proBNP	LVEF: 54.2±11.1%<br>Overall survival during the six months: 95.0%<br><br>Cardiac rehabilitation was associated with reduced mortality (OR 0.31; 95% CI 0.14–0.71; P = 0.006)	LVEF: 52.5±11.7%<br>Overall survival during the six months: 89.8%
						TAVR	SAVR
Imran et al.[13]	At the beginning:<br><br>Aerobic session: arm bike, recumbent bike, and/or Nustep<br>Low intensity, 70%-85% of HRmax achieved during entrance test or Rate of Perceived Exertion Scale<br>Exercise duration and intensity	Stress and mood management	Nutrition advice	Education on exercise	AACVPR category<br>GAD-7<br>SF-36<br>PHQ-9<br>ENRICHD survey<br>RYP survey<br>PANAS survey<br>ESSI	Exercise duration: 20.1±6.5 to 34.1±5.5min<br>Exercise intensity (6MWT): 2.6±0.9 to 3.6±1.3METS<br>Depression score	Exercise duration: 25.3±5.9 to 36.0±7.4min<br>Exercise intensity (6MWT): 3.3±1.0 to 4.6±1.6METS<br>Anxiety score (GAD-

© 2025. Han XS et al. Published online at http://www.aginganddisease.org/EN/10.14336/AD.2025.0716

SUPPLEMENTARY DATA

	increased on a weekly basis<br>90min/time, 3 times/week<br><br>During weeks 3-5:<br><br>Low resistance exercises:<br>Resistance gradually increased<br>90min/time, 3 times/week<br><br>Higher intensity aerobic exercise or another exercise modality<br>90min/time, 3 times/week<br><br>During weeks 6-12:<br><br>Exercise intensity increased<br>90min/time, 3 times/week				Symptom-limited exercise test<br>6MWT	(PHQ-9): 4.6±3.6 to 2.3±2.0<br>Positive affect (PANAS): 32.1±5.8 to 35.7±5.0<br>Negative affect (PANAS): 15.3±4.0 to 14.0±4.1<br>MCS (SF-36): 49.4±9.3 to 57.6±5.4	7): 2.3±3.4 to 1.5±2.4<br>Depression score (PHQ-9): 3.1±3.5 to 1.8±2.5<br>PCS (SF-36): 37.1±9.43 to 45.8±10.5
Rogers et al.[14]	Moderate interval intensity, progressively increased based on the self-reported BORG intensity<br>1 time/week, 6 weeks, 60-90 min/time<br><br>Graduated cardiovascular exercise: treadmill and bike<br><br>Resistance exercise<br><br>Functional exercise: sit to stand	—	—	—	LVEF<br>6MWT<br>Nottingham EADL<br>Fried Frailty score<br>EFS<br>HADS<br>KCCQ	CG	IG
						3 months<br>6MWT: -50.33 (-171.1, 70.4)<br>Nottingham EADL: 0.4 (-1.4, 2.1)<br>EFS: -0.1 (-1.7, 1.4)<br>HADS Anxiety: 0 (-3, 2)<br>HADS Depression: 1 (-2, 3)<br>6 months<br>6MWT: -92.6 (-206.7, 21.4)<br>Nottingham EADL: 0.7 (-1.5, 2.8)<br>EFS: 0.6 (-1.2, 2.3)<br>HADS Anxiety: 0 (-2, 2)<br>HADS Depression: 1 (0, 3)	
Genta et al.[15]	Aerobic session: cycling or treadmill exercise<br>Minimun workload to a value of 14 on the Borg Rating of Perceived Exertion Scale<br>30 min/time, 2 times/day (except for Saturday, only in the morning), 6 days/week, 3 weeks	—	Dietary counseling	Educational counseling	Echocardiography<br>BI<br>MFS<br>6MWT<br>CIRS-CI	Before CR	After CR
						BI: 62.1±24.5 to 83.04±21.2<br>6MWT: 175.6±80 to 238.3±76m	

© 2025. Han XS et al. Published online at http://www.aginganddisease.org/EN/10.14336/AD.2025.0716

SUPPLEMENTARY DATA

	If temporarily contraindicated treadmill or cycling exercise:<br>Walking along the 6MWT route:<br>30min/time, 1 time/day, every day<br><br>Pedal exercise:<br>0W<br>Up to 30 min/time, 2 times/day, every day<br><br>Respiratory session:<br>40min/time, 1 time/day, every day						
	If bedridden or markedly disabled or with a high risk of falls at admission:<br>Individualized physical therapy interventions for early mobilization<br>Followed the above described training protocol when their condition improved						
Bhattal et al.[16]	Total exercise:<br>2 hours/day, 12weeks	Stress management<br>Psychosocial consultation	Education about a healthy diet, food preparation, and logging intake of food	Smoking cessation	DASI<br>SF-36<br>Life's Simple 7 survey<br>6MWT	Before CR	After CR
	Aerobic exercise:<br>Walking/pedding<br>Low intensity, processed as tolerated<br>5 min/time, 3 days/week						
	Resistance exercise:<br>Stretching/TheraBands<br>3–5 min or 5–10 reps/time, processed as tolerated						
	Balance exercise						
Penati et al.[17]	Aerobic session:<br>Deambulatory type with intermittent mode (assessed by the SPPB scale)<br>Continuous aerobic exercise with the cycle ergometer (assessed by means of a 6MWT)<br>Low intensity (Borg 10/20)<br>30min/time, 1time/day, 6 days/week<br>only one of the two exercise on the sixth day	—	—	—	BI<br>6MWT<br>SPPB<br>Albumin<br>Hb value	Before CR	After CR
						BI: 73.80±23.31 to 90.22±16.53<br>6MWT: 265.43±89 to 327.17±111m<br>SPPB: 4.56±2.27 to 7.13±3.08	
	Group session:<br>Muscle strengthening, 10 repetitions for 2 of 10 different exercises<br>30%-50% of 1RM<br>Stretching with balance exercises and coordination<br>30min/time, 1time/day, 6 days/week						

© 2025. Han XS et al. Published online at http://www.aginganddisease.org/EN/10.14336/AD.2025.0716

	only one of the two exercise on the sixth day						
Lindman et al.[18]	Aerobic exercise:<br>Encourage at least 250 steps/h<br><br>Resistance exercises: 5–10 chair sit-to-stand, 5–10 chair push-ups, and 10 stress ball squeezes<br>1-5 times/day, 6 days/week, 6 weeks	—	—	—	SPPB<br>KCCQ<br>PROMIS 10<br>PROMIS CAT<br>Balance score<br>6MWT	IG	CG
						Daily steps: 1730 (100, 3360)<br>Daily active minutes: 66 (28, 105)<br>Moderate-to-intense active minutes: 53 (27, 80)<br>Sedentary minutes: -157 (-265, -50)	
Tamulevičiūtė-Prascienė et al.[19]	Exercise training (IG and CG):<br><br>Aerobic endurance training:<br>Low to moderate intensity, ≤30–50% maximal watt or 60–70% maximal HR<br>6 days/week, 40min/day<br><br>Aerobic dynamic gymnastics:<br>Low to moderate intensity,≤13 BORG<br>5 days/week, 30 min/day<br><br>Respiratory muscle training:<br>10 repetitions/session, few times /day<br>7 days/week, 15 min/day	Psychological support	Diet counselling	Patient education<br>Risk factor management	NYHA-class<br>Echocardiography<br>6MWT<br>SPPB<br>5MWT<br>CPET<br>1RM for leg extension	IG	CG
						T0 to T1:<br>6MWT: 247±94.1 to 348±100.1m<br>SPPB: 8.31±2.21 to 9.51±2.24<br>5MWT: 0.847±0.31 to 0.965±0.32m/s<br>Work load: 70.5±24.9 to 76.4±26.7 watt<br>Work load: 0.94±0.30 to 1.02±0.32 watt/kg<br>1RM for leg extension: 46.6±17.5 to 59.5±21.5kg<br>Frail (SPPB): 40.8% to 28.6%<br>Frail (5MWT): 40.4% to 21.7%	T0 to T1:<br>6MWT: 232±102.8 to 333±120.7m<br>SPPB: 7.95±2.01 to 9.08±2.35<br>5MWT: 0.765±0.24 to 0.879±0.29m/s<br>Work load: 67.8±16.6 to 81.8±24 watt<br>Work load: 0.85±0.17 to 1.04±0.27 watt/kg<br>1RM for leg extension: 50.5±20.9 to 53.5±21.1kg<br>Frail (SPPB): 37.8% to 29.7%<br>Frail (5MWT): 41.7% to 30.6%
	Additional resistance and balance training, 3 times/week (IG):					T0 to T1 to T2:<br>6MWT: 251±9.1 to	T0 to T1 to T2:<br>6MWT: 260±106.4

© 2025. Han XS et al. Published online at http://www.aginganddisease.org/EN/10.14336/AD.2025.0716

		First week, intensity BORG 11 and < 30% RM:<br><br>Balance training<br>15 min/day<br><br>Strength training<br>3 sets with 5–10 repetitions, with 30 s rest between sets<br>20–25 min/day					366±104.0 to 348±113.1m<br>SPPB: 8.47±2.37 to 9.8±2.3 to 10.37±1.59<br>5MWT: 0.907±0.341 to 1.049±0.392 to 1.086±0.307m/s<br>Peak work load: 70.3±25.9 to 78.1±27 to 93.6±42.8 watt<br>Peak work load: 1.0±0.4 to 1.1±0.3 to 1.4±0.5 watt/kg<br>1RM for leg extension: 46.6±17.5 to 59.5±21.5 to 60.1±22.2kg<br>Frail (SPPB): 36.7% to 26.7% to 6.6%<br>Frail (5MWT): 33.3% to 12.12% to 3.3%<br>PeakVO2: 12.8±5.6 to 13.3±3.5 to 15.8±4.7 ml/kg/min	to 357±123.3 to 332±147.4m<br>SPPB: 8.56±2.03 to 9.00±2.37 to 9.44±2.34<br>5MWT: 0.761±0.267 to 0.878±0.321 to 1.123±0.539m/s<br>Peak work load: 71.5±17.3 to 87.0±25.5 to 91.8±21.7 watt<br>Peak work load: 0.910±0.3 to 1.089±0.3 to 1.206±0.34 watt/kg<br>1RM for leg extension: 50.5±20.9 to 53.5±21.1 to 54.5±22.2kg<br>Frail (SPPB): 25% to 31.3% to 12.5%<br>Frail (5MWT): 43.8% to 31.3% to 12.5%<br>PeakVO2: 11.6±2.9 to 13.3±3.5 to 13.8±4.7 ml/kg/min	
		Second week, intensity BORG 12–13 and 30–50% RM:<br><br>Balance training<br>15 min/day<br><br>Strength training<br>3 sets with 15 repetitions, with 60 s rest between sets,<br>20–25 min/day							
		Third week, intensity BORG ≤15 and 50–60% RM:<br><br>Balance training,<br>15 min/day<br><br>Strength training<br>3 sets with 15 repetitions, with 60 s rest between sets<br>20–25 min/day							
	Hu et al.[20]	MICT: cycle and fast walking<br>Determined by the heart rate at anaerobic threshold measured by the CPET<br>Starting at a minimum intensity, gradually increasing to the maximum intensity every month<br>3 times/week, 3 months	—	—	—	CPET<br>6MWT<br>SF-12<br>Echocardiographic parameters<br>Laboratory parameters	MICT	Control	
							Peak VO_2: 1.63 mL/kg/min (0.58, 2.67)<br>MET: 0.51 (0.21, 0.81)<br>VO_2AT: 1.84 mL/kg/min (1.07, 2.62)<br>METAT: 0.55 (0.32, 0.78)<br>6MWT: 21.55 (0.38, 42.71)<br>LDL cholesterol: -0.62 mmol/L (-1.00, -0.23)		
		Warm-up:<br><50% target intensity for 2min, gradually increasing the load 1 to 10 W/min up to 100% target intensity for 5-10 min							

© 2025. Han XS et al. Published online at http://www.aginganddisease.org/EN/10.14336/AD.2025.0716

	Exercise phase:<br>100% target intensity<br>Starting with 20 minutes and gradually lengthening up to 45 minutes in the 1 month or maintaining for 45 minutes in the following 2 months					
	Cool down:<br>Gradual reduction of load<br>Within 3 minutes					

CR: cardiac rehabilitation; TAVR: Transcatheter aortic valve replacement; SAVR: Surgical aortic valve replacement; NYHA: New York Heart Association; FIM: Functional independence measure; HADS: Hospital Anxiety and Depression Scores; 6MWT: 6-min walk test; 5MWT: 5 m walk time; HGS: hand grip strength; KI: Katz index; ADL: Activities of Daily Living; KCCQ: Kansas City Cardiomyopathy Questionnaire; DH: discharged home; STS: sit-to-stand; mMRC: modified Medical Research Council; DASI: Duke Activity Status Index; PSQI: Pittsburgh Sleep Quality Index; BI: Barthel Index; FS-14: Fatigue Scale-14; SF-12: 12-Item Short-Form Health Survey; IMT: Inspiratory muscle training; FEV_1: forced expiratory volume in one second; MIP: Maximal inspiratory pressure; BADL: basic activities of daily living; MMSE: Mini Mental State Examination; MNA: Mini Nutritional Assessment; MET: metabolic equivalent; EQ-5D: EuroQol five dimension scale; GDS: Geriatric Depression Scale; CIRS-CI: Cumulative Illness Rated State-Comorbidity Index; MUST: Malnutrition Universal Screening Tool; EQ VAS: EQ visual analogue scale; HR: heart rate; LVEF: Left ventricular ejection fraction; CPET: Cardiopulmonary exercise test; TG: training group; UC: usual care; VO_2peak: peak oxygen uptake; VO_2AT: oxygen anaerobic threshold; IADL: Instrumental Activities of Daily Living; TUG: Timed Up and Go; PCS: physical component scales; MCS: mental component scales; MFS: Morse Fall Scale; AACVPR: American Association of Cardiovascular and Pulmonary Rehabilitation; GAD-7: General Anxiety Disorder-7; SF-36: 36-Item Short-Form Health Survey; PHQ-9: Patient Health Questionnaire-9; ENRICHD: Enhancing Recovery in Coronary Heart Disease Patients; RYP: Rate Your Plate; PANAS: Positive and Negative Affect Schedule; ESSI: ENRICHD Social Support Inventory; CG: control group; IG: intervention group; EADL: Extended Activities of Daily Living; EFS: Edmonton Frailty score; SPPB: Short physical performance battery; PROMIS: Patient-Reported Outcomes Measurement Information System; CAT: computerized adaptive test; RM: repetition maximum; MICT: Moderate Intensity Continuous Training

References

[1] Fauchere I, Weber D, Maier W, Altwegg L, Luscher TF, Grunenfelder J, *et al.* (2014). Rehabilitation after TAVI compared to surgical aortic valve replacement. Int J Cardiol, 173:564-566.

[2] Kleczynski P, Trebacz J, Stapor M, Sobczynski R, Konstanty-Kalandyk J, Kapelak B, *et al.* (2021). Inpatient Cardiac Rehabilitation after Transcatheter Aortic Valve Replacement Is Associated with Improved Clinical Performance and Quality of Life. J Clin Med, 10:2125.

[3] Xu L, Wei J, Liu J, Feng Y, Wang L, Wang S, *et al.* (2023). Inspiratory muscle training improves cardiopulmonary function in patients after transcatheter aortic valve replacement: a randomized clinical trial. Eur J Prev Cardiol, 30:191-202.

[4] Yu Z, Zhao Q, Ye Y, Wang M, Zhou Z, Zhang H, *et al.* (2021). Comprehensive Geriatric Assessment and Exercise Capacity in Cardiac Rehabilitation for Patients Referred to Transcatheter Aortic Valve Implantation. Am J Cardiol, 158:98-103.

[5] Zanettini R, Gatto G, Mori I, Pozzoni MB, Pelenghi S, Martinelli L, *et al.* (2014). Cardiac rehabilitation and mid-term follow-up after transcatheter aortic valve implantation. J Geriatr Cardiol, 11:279-285.

[6] Russo N, Compostella L, Tarantini G, Setzu T, Napodano M, Bottio T, *et al.* (2014). Cardiac rehabilitation after transcatheter versus surgical prosthetic valve implantation for aortic stenosis in the elderly. Eur J Prev Cardiol, 21:1341-1348.

[7] Völler H, Salzwedel A, Nitardy A, Buhlert H, Treszl A, Wegscheider K (2015). Effect of cardiac rehabilitation on functional and emotional status in patients after transcatheter aortic-valve implantation. Eur J Prev Cardiol, 22:568-574.

[8] Pressler A, Christle JW, Lechner B, Grabs V, Haller B, Hettich I, *et al.* (2016). Exercise training improves exercise capacity and quality of life after transcatheter aortic valve implantation: A randomized pilot trial. Am Heart J, 182:44-53.

[9] Eichler S, Salzwedel A, Reibis R, Nothroff J, Harnath A, Schikora M, *et al.* (2017). Multicomponent cardiac rehabilitation in patients after transcatheter aortic valve implantation: Predictors of functional and psychocognitive recovery. Eur J Prev Cardiol, 24:257-264.

[10] Tarro Genta F, Tidu M, Bouslenko Z, Bertolin F, Salvetti I, Comazzi F, *et al.* (2017). Cardiac rehabilitation after transcatheter aortic valve implantation

© 2025. Han XS et al. Published online at http://www.aginganddisease.org/EN/10.14336/AD.2025.0716

compared to patients after valve replacement. J Cardiovasc Med (Hagerstown), 18:114-120.
[11] Pressler A, Förschner L, Hummel J, Haller B, Christle JW, Halle M (2018). Long-term effect of exercise training in patients after transcatheter aortic valve implantation: Follow-up of the SPORT:TAVI randomised pilot study. Eur J Prev Cardiol, 25:794-801.
[12] Butter C, Groß J, Haase-Fielitz A, Sims H, Deutsch C, Bramlage P, *et al.* (2018). Impact of Rehabilitation on Outcomes after TAVI: A Preliminary Study. J Clin Med, 7:326.
[13] Imran HM, Baig M, Mujib M, Beale C, Gaw A, Stabile L, *et al.* (2018). Comparison of phase 2 cardiac rehabilitation outcomes between patients after transcatheter versus surgical aortic valve replacement. Eur J Prev Cardiol, 25:1577-1584.
[14] Rogers P, Al-Aidrous S, Banya W, Haley SR, Mittal T, Kabir T, *et al.* (2018). Cardiac rehabilitation to improve health-related quality of life following trans-catheter aortic valve implantation: a randomised controlled feasibility study: RECOVER-TAVI Pilot, ORCA 4, For the Optimal Restoration of Cardiac Activity Group. Pilot Feasibility Stud, 4:185.
[15] Tarro Genta F, Tidu M, Corbo P, Bertolin F, Salvetti I, Bouslenko Z, *et al.* (2019). Predictors of survival in patients undergoing cardiac rehabilitation after transcatheter aortic valve implantation. J Cardiovasc Med (Hagerstown), 20:606-615.
[16] Bhattal GK, Park KE, Winchester DE (2020). Home-Based Cardiac Rehabilitation (HBCR) In Post-TAVR Patients: A Prospective, Single-Center, Cohort, Pilot Study. Cardiol Ther, 9:541-548.
[17] Penati C, Incorvaia C, Mollo V, Lietti F, Gatto G, Stefanelli M, *et al.* (2021). Cardiac rehabilitation outcome after transcatheter aortic valve implantation. Monaldi Arch Chest Dis, 91.
[18] Lindman BR, Gillam LD, Coylewright M, Welt FGP, Elmariah S, Smith SA, *et al.* (2021). Effect of a pragmatic home-based mobile health exercise intervention after transcatheter aortic valve replacement: a randomized pilot trial. Eur Heart J Digit Health, 2:90-103.
[19] Tamulevičiūtė-Prascienė E, Beigienė A, Thompson MJ, Balnė K, Kubilius R, Bjarnason-Wehrens B (2021). The impact of additional resistance and balance training in exercise-based cardiac rehabilitation in older patients after valve surgery or intervention: randomized control trial. BMC Geriatr, 21:23.
[20] Hu Q, Li Y-S, Ren Q, Liang Y-C, Zhang J, Wang Y-X, *et al.* (2023). Efficacy and Safety of Moderate-Intensity Continuous Training on the Improvement of Cardiopulmonary Function in Patients After Transcatheter Aortic Valve Replacement (ENERGY): A Randomized Controlled Trial. Journal of the American Medical Directors Association, 24:1783-1790.e1782.

© 2025. Han XS et al. Published online at http://www.aginganddisease.org/EN/10.14336/AD.2025.0716

SUPPLEMENTARY DATA

© 2025. Han XS et al. Published online at http://www.aginganddisease.org/EN/10.14336/AD.2025.0716
